# Polypharmacy-associated risk of hospitalisation among people ageing with and without HIV: an observational study

**DOI:** 10.1016/S2666-7568(21)00206-3

**Published:** 2021-09-29

**Authors:** Amy C Justice, Kirsha S Gordon, Jonathon Romero, E Jennifer Edelman, Benjamin J Garcia, Piet Jones, Saye Khoo, Vincent Lo Re, Christopher T Rentsch, Janet P Tate, Alice Tseng, Julie Womack, Daniel Jacobson

**Affiliations:** School of Medicine, Yale University, New Haven, CT, USA (Prof A C Justice MD, K S Gordon PhD, E J Edelman MD, J P Tate ScD); VA Connecticut Healthcare System, West Haven, CT, USA (Prof A C Justice, K S Gordon, J P Tate, C T Rentsch PhD, J Womack PhD); Bredesen Center for Interdisciplinary Research and Graduate Education, University of Tennessee, Knoxville, TN, USA (J Romero BSc, P Jones MSc); Biosciences Division, Oak Ridge National Laboratory, Oak Ridge, TN, USA (B J Garcia PhD, D Jacobson PhD); Department of Molecular and Clinical Pharmacology, University of Liverpool, Liverpool, UK (Prof S Khoo MD); Department of Medicine, Perelman School of Medicine, University of Pennsylvania, Philadelphia, PA, USA (V Lo Re III MD); Faculty of Epidemiology and Population Health, London School of Hygiene & Tropical Medicine, London, UK (C T Rentsch); University Health Network and Faculty of Pharmacy, University of Toronto, Toronto, ON, Canada (A Tseng PharmD); Faculty of Yale University School of Nursing, West Haven, CT, USA (J Womack)

## Abstract

**Background:**

Polypharmacy, defined as use of five or more medications concurrently, is associated with adverse health outcomes and people ageing with HIV might be at greater risk than similar uninfected individuals. We aimed to determine whether known pairwise drug interactions (KPDIs) were associated with risk of admission to hospital (hereafter referred to as hospitalisation) and medication count among people ageing with and without HIV after accounting for physiological frailty.

**Methods:**

In this observational study, we collected individual-level data for participants of the Veterans Aging Cohort Study (VACS) with HIV on antiretroviral therapy (ART) and with supressed HIV-1 RNA and people without HIV who were receiving at least one prescription medication, based on active medications in the 2009 fiscal year (ie, Oct 1, 2008, to Sept 30, 2009). We identified KPDIs among these patients by linking prescription fill and refill data with data from DrugBank (version 5.0.11). We collected data on all-cause mortality and hospitalisations between Oct 1, 2009, and March 31, 2019. We compared KPDI counts using random selection and actual patterns of use across medication counts from two to 12. We created a weighted KPDI Index on the basis of the average association of each KPDI with mortality among people ageing without HIV and used nested Cox models stratified by HIV status to estimate the association between medication count and hospitalisation, with incremental adjustments for demographics, physiological frailty, and KPDI Index.

**Findings:**

We collected data for 9186 people ageing with HIV and 37 930 individuals without HIV. 45 913 (97·4%) of 47 116 patients were men and the sample was predominantly aged 50–64 years (30 413 [64·6%]). Compared with a random sample of medications, real-world pattern of medication counts and combinations were associated with five-to-six times more KPDIs (eg, for a combination of six medications, KPDI count was 1·09 in the random sample, 5·49 in the HIV-negative population, and 7·13 in the HIV-positive population). For each additional observed medication, people ageing with HIV had approximately 2·94 additional KPDIs and comparators had approximately 2·67 additional KPDIs. Adjustment for demographics, physiological frailty, and KPDI Index reduced the association between medication count and risk of hospitalisation for people ageing with HIV (hazard ratio 1·08 [95% CI 1·07–1·09] reduced to 1·06 [1·05–1·07]) and those without HIV (1·08 [1·07–1·08] reduced to 1·04 [1·03–1·05]).

**Interpretation:**

For each additional medication, people ageing with HIV have more drug–drug interactions than those without HIV. Adjusting for known non-ART drug–drug interactions, each additional non-ART medication confers excess risk of hospitalisation for people ageing with HIV. Randomised trials will be needed to determine whether reducing these interactions improves outcomes.

**Funding:**

National Institutes of Health, National Institute on Alcohol Abuse and Alcoholism, Department of Veterans Affairs Health Services Research & Development, and Office of Research and Development.

## Introduction

Polypharmacy is the simultaneous use of multiple medications (often five or more)^1^ and is widespread among older adults in North America and Europe.^2,3^ Polypharmacy is associated with non-adherence,^4^ adverse drug events,^5^ falls,^6^ opioid overdose,^7^ and adds substantial complexity to medical regimens, thereby contributing to inappropriate prescribing.^8^ It is also associated with admission to hospital (hereafter referred to as hospitalisation)^9^ and mortality^1^ in a dose-response manner.

**Figure 1:**
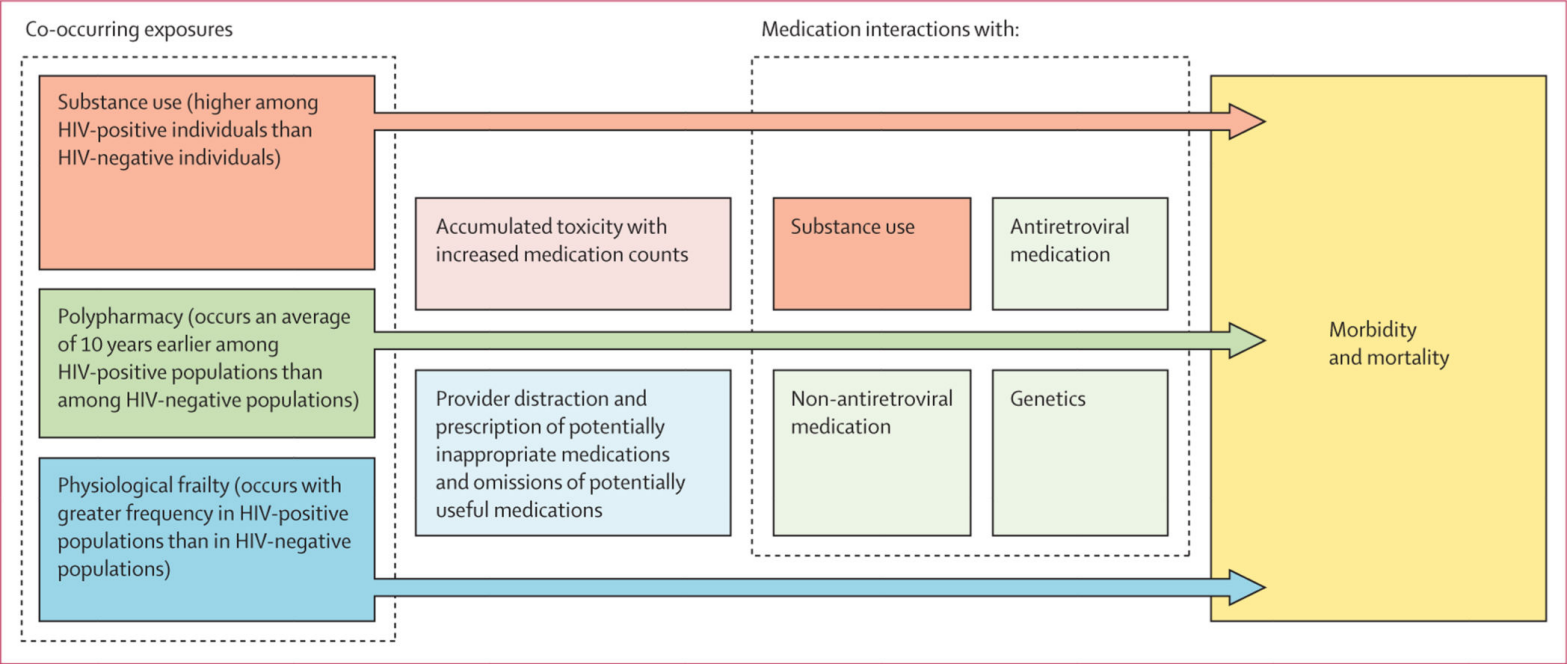
Mechanisms of harm from polypharmacy for people ageing with HIV compared with those without HIV

Polypharmacy might be particularly problematic for people ageing with HIV. People ageing with HIV might be more susceptible to adverse effects from specific medications and to cumulative injury from polypharmacy than demographically similar uninfected individuals (figure 1).^10^ Although antiretroviral therapy (ART) is life preserving, it must be sustained for a patient’s lifetime and typically includes three active medications. People ageing with HIV typically have polypharmacy a decade earlier than uninfected individuals.^11^ Furthermore, compared with demographically similar uninfected individuals, people ageing with HIV have excess physiologial frailty,^12^ making them more susceptible to the adverse effects of medications. An increased number of medications being taken concomitantly increases the probability of harmful interactions with other medications, alcohol, and other substances, and with individuals’ differing pharmaco genomics.^7,13,14^

ART medications commonly interact with non-ART medications.^15^ For example, boosted regimens, including protease inhibitors and the early integrase strand transfer inhibitor elvitegravir–cobicistat, can increase drug concentrations and risk of adverse effects of co-medications via inhibition of CYP3A4 and transporters.^16^ Interactions between ART and non-ART medications are associated with hospitalisation after adjusting for CD4 cell count, protease inhibitor-based regimen, substance use, and non-ART medication count.^17^ While now being prescribed less frequently, use of boosted regimens remains common.^18^ To date, no study has considered the association between hospitalisation and all pairwise drug interactions including both ART–non-ART and non-ART–non-ART medication interactions among people ageing with HIV.

The greatest limitation for observational studies of polypharmacy is confounding by indication (individuals who are sicker or more frail are on more medications).^19^ One approach to partially address this confounding is to adjust for physiological frailty. Based on routine clinical laboratory data, the Veterans Aging Cohort Study (VACS) Index (version 1.0) has been validated as a highly discriminating and generalisable measure of physiological frailty in people ageing with HIV and people ageing without HIV.^12^

We previously found in a large national study done in the USA that among people with and without HIV, that risk of hospitalisation and mortality increased with increasing medication count after adjustment for VACS Index 1.0 score.^20^ Our findings suggested that confounding by indication alone did not explain the association between polypharmacy and adverse health outcomes. Subsequently, we have developed and validated an even more discriminating measure of physiological frailty (VACS Index version 2.0).^21^ In the current study, we aimed to further enhance our adjustment for physiological frailty using VACS Index 2.0.

Finally, the potential list of drug interactions is large and highly complex, but known pairwise drug interactions (KPDIs) have been meticulously catalogued in a centralised database, DrugBank.^22^ DrugBank includes KPDI information for US Food and Drug Association-approved drugs. DrugBank version 5.0.11 provides a catalogue of 365 984 evidence-based KPDIs. Linking these data to a clinical cohort and analysing associations required innovative methods. Although similar databases exist, DrugBank attempts to be as comprehensive as possible and was updated in 2018.

To better understand mechanisms underlying harms due to polypharmacy among people ageing with and without HIV infection, we added DrugBank data, created a KPDI Index, extended follow-up, and updated our physiological frailty index (VACS Index 2.0) to extend our previous study.^20^ Here, we explored the association between KPDI and medication count, considering both ART and non-ART medications; determined the degree to which KPDIs drive the association between non-ART medication count and hospitalisation, test whether associations differ by HIV status; and determine how much risk of hospitalisation associated with non-ART medication count remains after better accounting for physiological frailty and KPDIs.

## Methods

### Study design and population

In this observational study, we included people ageing with HIV receiving ART who had suppressed HIV-1 RNA (ie, ≤400 copies per mL) and people without HIV who were receiving at least one prescription medication from the US Veterans Affairs Healthcare System (VA) in the fiscal year of 2009 (Oct 1, 2008, to Sept 30, 2009). Briefly, the VACS is a longitudinal, multicentre, observational study of HIV infected and uninfected individuals who receive care in the national VA health-care system in the USA. Enrolment began in 1999 and additional patients are added and followed up each year.

For this analysis, patients were followed up from Oct 1, 2009, until March 31, 2019, for hospitalisation and all-cause mortality. Cohort characteristics, measurement of medication exposure, and outcomes are the same as in the original publication.^20^ More details of our methods are provided in the appendix (pp 1–4).^20^

As in the original study,^20^ we restricted our analysis to medications filled on a chronic basis, defined as at least 90 consecutive days allowing for a 30 day refill window, consistent with previous definitions.^23^ These data were extrapolated from pharmacy data from the VA. We calculated days of receiving medication on the basis of medication fill refill information, assuming the medication was taken as directed. Patients might have started a medication before the 2009 fiscal year or continued them after the fiscal year; we only captured days supplied within this fiscal year. We counted each component of co-formulated medications separately. In this analysis, we included longer-term follow-up and use VACS Index 2.0 rather than VACS Index 1.0 for physiological frailty adjustment.

### VACS Index

We used the VACS Index 2.0 to adjust for frailty.^12,21^ The VACS Index 1.0 and 2.0 are indices of physiological frailty that can be used to predict the risk of all-cause mortality and were initially developed using a cohort of people ageing with HIV. VACS Index 1.0 integrates data on age, HIV biomarkers (HIV-1 RNA viral load, CD4 cell count), and non-HIV biomarkers (haemoglobin, hepatitis C, Fibrosis-4 [known as FIB-4] score to assess liver function, and estimated glomerular filtration rate to assess renal function). VACS Index 2.0 improves on the discrimination observed using VACS Index 1.0 by adding body-mass index, albumin, white blood cell count, and continuous functional forms for all variables. Neither index includes HIV status, thereby enabling application to uninfected individuals. Both indices include viral load level (assumed 0 in HIV-negative individuals) and CD4 cell count (assumed to be greater than 500 cells per mm^3^; in HIV-negative individuals). The VACS Index has been validated in HIV-negative individuals.^12^ Scores typically range from 0 to 100, with higher scores indicating a greater risk of mortality from physiological injury. A five-point increase in VACS Index 2.0 score is associated with a 30% increase in 5 year mortality risk.^21^ We determined scores using laboratory values closest to the end of the 2009 fiscal year. The requirement of the additional variables for the VACS Index 2.0 versus 1.0 slightly reduced our sample size from that in our original analyses (from 9473 patients ageing with HIV in the original analysis to 9186 and people ageing without HIV from 39 812 to 37 960).^20^

### DrugBank mapping, Drug Interaction Network, and co-occurrence matrix

We parsed the DrugBank (version 5.0.11) database^22^ for drug interactions between medications taken by the cohort. We initially mapped drugs to synonyms and base terms in the DrugBank database. Any drugs that did not have an automatic mapping to DrugBank were manually assessed to determine if there was a matching DrugBank term. All drugs without a matching term in DrugBank were excluded. Drugs dispensed from the pharmacy were further split into subsets by removing herbal and dietary supplements (eg, fish oil, vitamins and minerals, peppermint oil), vaccines, water, non-specific skin ointments and lubricants, and other non-specific terms. Matching, subsetting, and exclusion resulted in a reduction of 159 drugs (from 709 to 550).

We then created a Drug Interaction Network from the drugs within the database (nodes) and KPDIs (edges). The resulting network was visualised with Cytoscape (version 3.80).^24^ We created a calendar for each patient, mapping start and stop times for each drug to their respective dates. All days in between the start and stop date were counted as the days a patient was on a given medication. Two or more medications were said to have an overlap if a given patient was taking both or each drug on at least 1 day in common. We removed duplicates of medication overlaps to give us a set of unique co-medications for each patient. We then created a matrix (called the Drug Co-Occurrence Matrix) using all the co-medications for all patients.

### Simulated drug set interactions

We first considered KPDIs that might occur in theory, by chance alone. We did this by writing a Perl program to generate random drug sets for a population size of 44 350 individuals. This number of individuals is less than the entire sample because an individual must be on at least two medications to have a drug–drug interaction. Specifically, 44 350 drug sets were generated randomly for each set of medications from two to 12 (including ART medications). Each set was used in conjunction with the Drug Interaction Network to determine how many known drug interactions were contained within each set. Briefly, all possible pairs of drugs within the DrugBank database were generated and the Drug Interaction Network queried for the presence of an interaction edge between each pair of drugs. The minimum, maximum, and mean number and SD of drug interactions were calculated across all sets for each set size. To test the effect of population size on this simulation, we did a similar random drug interaction simulation on a population size of 20 000 000 individuals and found that the results were not sensitive to this difference in population size. Results are shown from the smaller sample in the Results section.

Then, we wrote another Perl program to generate drug sets from the Drug Co-Occurrence Matrix. These sets were then used in conjunction with the Drug Interaction Network to determine how many KPDIs were contained within each set. Briefly, all observed pairs of drugs for each patient were generated and the Drug Interaction Network queried for the presence of an interaction edge between each pair of drugs. The minimum, maximum, and mean number and SD of drug interactions were calculated across the sets for each set size from two to 12 (including ART medications).

### KPDI Index

We sought to develop a summary weighted measure of KPDIs on the basis of the average association of each KPDI with mortality among individuals who were HIV negative. Using mortality as the outcome event and focusing on HIV-negative individuals, we trained a Cox proportional hazards model to create a beta coefficient for each KPDI. We focused on HIV-negative individuals to weight the interactions in the largest subset, without the effects of HIV infection and antiretrovirals. We did this with a program, written in Python, using the Drug Co-occurrence Matrix. Drug interactions with fewer than ten instances were removed due to low prevalence in the population.

To mitigate overfitting, we used a technique called bagging that is commonly used in machine learning, in which the same calculation is done on different subsets of the data (ie, different bags) hundreds of times. The resulting average of the different bags is more robust to sample bias and produces more accurate predictors.^25^ The dataset was sampled with replacement for 1000 iterations producing subsets that contained the same number of total samples and for each iteration a model was built for each KPDI. Subsets with hazard ratio (HR)-associated p values of more than 0·05 were assigned an HR of 1·0. And the results of each KPDI with an HR with a p value of less than 0·05 were averaged across the iterations for each KPDI and a 95% CI for the HR was calculated for each.

We then used these estimates to create a KPDI Index, weighted by the period of exposure to each KPDI within the year of observation. We did this by summing the beta coefficients associated with each KPDI across all KPDIs to which the patient was exposed to generate a KPDI Index for each patient on the log hazard scale. We analysed this index as both a continuous measure and separated into ranked quintiles with approximately equal numbers of death (among the uninfected comparators) occurring in each quintile. Fully adjusted models estimating HRs for medication count in which the KPDI is treated as an indicator variable rather than as a continuous measure had a superior model fit. Here, we show only the indicator models. The range of values for the KPDI Index estimated on those without HIV was from –5·6 to 30·7. Negative values correspond to a protective association with mortality, whereas higher positive values correspond to a greater risk of mortality.

Notably, the KPDI Index score for an individual drug pair from DrugBank measures the association the drug pair has to mortality, controlling for overall health, by incorporating the VACS Index 2.0 in the model. By bootstrapping across multiple subsampled sets, we ensured that only the most robust associations were considered to be significant. We used this method to ensure that both potentially spurious correlations and drug pairs with low effect have low-to-zero weight in the final model.

### Additional statistical analyses

We did a descriptive analysis of the study sample overall and by HIV status. We did an analysis of bivariate associations by randomly selecting among all medications included medication counts and comparing their association with KPDI to actual medication counts by HIV status. Because people ageing without HIV infection do not take ART, we only considered non-ART medications when considering medication count and when developing the KPDI Index. However, because ART medications interact with many non-ART medications, we considered both when calculating counts of drug–drug interactions. We used Pearson’s correlation to determine the association between medication count and VACS and KPDI Index score. We calculated unadjusted mortality and hospitalisation rates per 1000 person-years by dividing the number of observed events by the person-years of observation. We used nested Cox models stratified by HIV status to estimate the association between medication count and hospitalisation—incrementally adjusting for age per 10 years, sex, race, VACS Index score per 5 point increase , and KPDI Index score by quintile and risk of hospitalisation. In these analyses, we censored patients on date of death or, if alive at the end of follow-up, on March 31, 2019. In our nested model we adjusted for demographics, physiological frailty, and KPDI index to understand the relative explanatory power of each of these components for the overall association between medication count and hospitalisation. We assessed the proportional hazards assumption by including interactions of exposure variables with follow-up time. The interactions with medication count and with sex were not significant for those with and without HIV.

All p values of less than 0·05 were considered to be significant. We did all additional statistical analyses using SAS (version 9.4).

### Role of the funding source

The funder of the study had no role in study design, data collection, data analysis, data interpretation, or writing of the report.

## Results

During the 2009 fiscal year (baseline), 9186 people ageing with HIV in the VACS were on ART with suppressed HIV-1 RNA and 37 960 people ageing without HIV received at least one medication from the VA pharmacy (table 1). The cohort was predominantly aged 50–64 years (30 413 [64·6%] of 47 116), racially and ethnically diverse, male (45 913 [97·4%]), and demographically similar by HIV status. Both people ageing with HIV and people ageing without HIV were engaged in care, with the mean number of health-care visits per year among people ageing with HIV being 2·1 (SD 1·1) and in people ageing without HIV being 1·9 (1·0). Among people ageing with HIV, median CD4 count was 511 cells per mm^3^ (IQR 346–720). Consistent with our inclusion criteria, all people ageing with HIV were on ART (5613 [61·1%] of 9186 were on protease inhibitors, 3618 [39·4%] were on non-nucleoside reverse transcriptase inhibitors, and five [<0·1%] were on an integrase inhibitors) and had HIV-1 RNA concentrations of 400 copies per mL or lower. Overall, non-ART medication counts were lower for people ageing with HIV than those without HIV (mean count: 3 [SD 3] *vs* 4 [3]). People ageing with HIV had higher VACS Index 2.0 scores, suggesting greater physiological frailty than those without HIV (mean score: 50 [SD 15] *vs* 33 [11]). Hospitalisation rates were higher for people ageing with HIV (108·9 admissions [95% CI 106·1–111·8] per 1000 person-years) than those without HIV (87·5 admissions [86·3–88·7] per 1000 person-years). Mortality rates were also higher among people ageing with HIV (30·6 [95% CI 29·4–31·9] per 1000 person-years) than among those without HIV (26·6 [95% CI 26·0–27·2] per 1000 person-years).

**Figure 2:**
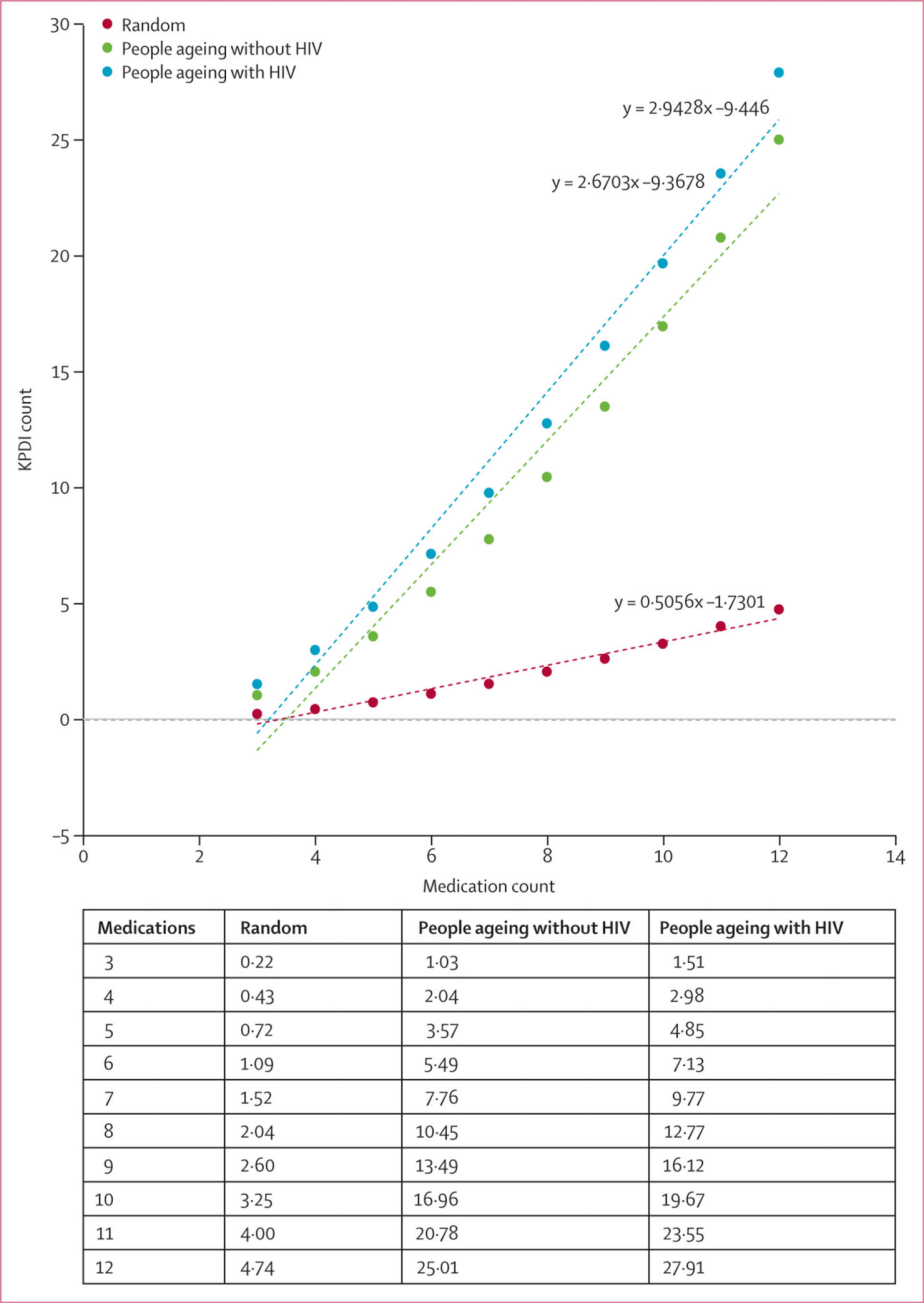
Theoretical and observed associations of medication count (including both ART and non-ART medications) and KPDIs Datapoints are estimates for each dataset at each medication count, with dotted lines joining these datapoints to show the linear association. ART=antiretroviral therapy. KPDI=known pairwise drug interaction.

When ART and non-ART medications were randomly selected, the association between medication count and KPDI count was linear (y = 0·51 x – 1·73; *R*^2^ = 0·97; figure 2). For each additional medication, random selection predicted an additional 0·51 KPDIs. When actual medications were considered by HIV status, the slope of the association was steeper but remained linear. For each additional medication, people ageing without HIV had an additional 2·67 KPDIs (y = 2·67 x – 9·38; *R*^2^ = 0·97) and people ageing with HIV had an additional 2·94 KPDIs (y = 2·94 x – 9·45; *R*^2^ = 0·98; figure 2). Medication count was weakly correlated with VACS Index 2.0 score (Pearson correlation 0·11 for both groups; data not otherwise shown).

People ageing with and without HIV with no KPDIs had low non-ART medication counts and higher all-cause mortality rates than those in the lowest KPDI Index quintile, even though their VACS Index scores were similar (table 2). People ageing with HIV and without HIV in the lowest KPDI Index quintile were taking two to three more medications than those with no KPDIs; however, they had lower mortality rates (22 *vs* 27 deaths per 1000 person-years for people ageing with HIV and 15 *vs* 17 deaths per 1000 person-years for people ageing without HIV) and similar VACS Index scores than those having no KPDIs. Although, KPDI Index score correlated with medication count overall (Pearson correlation 0·46; data not otherwise shown), we observed J-shaped associations between quintiles of KPDI Index score and non-ART medication count for both those ageing with and without HIV (table 2). People ageing with and without HIV in the second KPDI Index score quintile were taking the fewest non-ART medications of any quintile. This observation was also true for crude mortality rates, which were similar between KPDI Index quintile 1 and 2 for people ageing with HIV but were higher in quintile 2 than in quintile 1 for people ageing without HIV (table 2). However, VACS Index 2.0 scores were similar across quintiles 1 and 2 for both groups. Those in the KPDI Index quintiles 3, 4, and 5 were taking progressively more non-ART medications, had increasing VACS Index 2.0 scores, and had increasing rates of mortality regardless of HIV status (table 2).

In a series of nested models, we explored the association between non-ART medication count and risk of hospitalisation, stratified by HIV status (table 3). In unadjusted analyses, non-ART medication count was similarly associated with risk of hospitalisation among people ageing with HIV (HR 1·08 [95% CI 1·07–1·09]) and among those without HIV (1·08 [1·07–1·08]). Adjusting for demographics and physiological frailty (determined by VACS Index 2.0 score) resulted in a slight decrease in the association, similarly in people ageing with HIV (HR 1·07 [1·06–1·08]) and in those without HIV (1·07 [1·06–1·07]). Further adjusting for KPDI Index reduced the risk of hospitalisation associated with medication count for both people ageing with HIV (1·06 [1·05–1·07] and those without HIV (1·04 [1·03–1·05]).

## Discussion

Our study offers several new insights into the complex problem of polypharmacy and its implications for people ageing with and without HIV infection. First, across a range of commonly observed non-ART medication counts, KPDIs are five-to-six times more common in real-world data than if medications are randomly selected. This higher number of interactions is probably because medications that are given to address specific health conditions have overlapping mechanisms and adverse events. This was particularly true for people ageing with HIV, most likely because ART medications have an exceptionally large number of drug–drug interactions.^16,26^ Second, after adjustment for demographics and physiological frailty, the KPDI Index based on non-ART medications explained an important portion, but by no means all, of the remaining association between polypharmacy and risk of hospitalisation. After full adjustment, the remaining associations were stronger for people ageing with HIV than those without HIV. This finding was also true when the KPDI Index included ART medications. These results suggest that KPDIs are major contributors to morbidity for people ageing with and without HIV and that both groups are susceptible to harms due to polypharmacy that are not reflected in KPDIs alone. They also suggest that people ageing with HIV might be more susceptible to harm from polypharmacy than those without HIV, both due to KPDIs and other mechanisms of injury. Third, our results suggest that beneficial effects of individual medications might be diminished and possibly lost as the total count increases.

We previously showed that non-ART medication count has a strong dose–response association with mortality and hospitalisation among people ageing with HIV and demographically similar uninfected individuals.^20^ An independent association remained before and after adjustment for general physiological frailty using VACS Index 1.0.^20^ However, that analysis could not differentiate between mechanisms of injury. In the current study by applying data science techniques to DrugBank data in our real-world cohort, we were able to consider the role of KPDIs. We also applied VACS Index 2.0^12,21^ to more completely account for confounding by indication.

ART extends survival but must be taken for the rest of a patient’s life. Furthermore, ART initiation typically precipitates polypharmacy a full decade earlier for people ageing with HIV than those without HIV.^11^ ART medications interact with a wide range of common medications including lipid-lowering agents, antihypertensives, antidepressants, proton-pump inhibitors, and opioid agonists.^15,16^ However, no ready means of counting known drug–drug interactions, accounting for their differential effects, or comparing these by HIV status, has previously been available.

We were able to overcome these limitations using the DrugBank database combined with machine learning, simulation, and real-world data. Our simulation suggested, on average, an additional drug interaction for every two medications added. However, when we linked DrugBank data with real-world data we observed five-to-six times this number of interactions. Medications vary substantially in the number of other drugs with which they are known to interact. Our comparison of a random selection of drugs with real-world data underscores the nature of therapeutic medication selection; multiple medications are selected to treat specific conditions targeting specific mechanisms. Because medication targets can overlap, they are more likely to interact than if the medications were selected by chance.

By creating a KPDI Index weighted according to the average association of each pair with mortality, we accounted for this variation and optimised the ability of the KPDI Index to explain the association between non-ART medication count and adverse outcomes. When we summed pairwise interactions and their unadjusted component associations with survival into a severity index, we observed J-shaped associations with overall non-ART medication count and mortality rates. Although both people ageing with and without HIV infection in the lowest KPDI Index quintile were on more non-ART medications than those with no KPDI, they had lower mortality.

These associations suggest a benefit for a small number of medications, but this benefit was lost as the number of medications and total number of KPDIs increased among both people ageing with and without HIV. When an individual was on a large number of medications, the harmful effects of polypharmacy appeared to outweigh benefit. Previous work has often identified a threshold of five medications for adverse effects of polypharmacy.^1^ Our analyses suggest that medication counts as low as four might be problematic if associated with a high KPDI Index. However, if the KPDI Index is low, four or five medications might still be beneficial. Medication counts exceeding this threshold were associated with higher KPDI Index scores and crude mortality rates.

People ageing with HIV were on a lower number of non-HIV medications than those without ART, and their physiological frailty (as reflected in their VACS Index 2.0 scores) was higher. We suspect that HIV-care providers are already concerned about polypharmacy and drug–drug interactions, and are less likely to prescribe non-ART medications than general care providers. This finding is in contrast with studies that focused on polypharmacy among people ageing with HIV in which non-HIV and HIV care are provided in clinics that do not share a single electronic medical record. In these settings, people ageing with HIV had more polypharmacy than uninfected individuals,^18,27,28^ possibly because providers were unaware of medications prescribed in outside clinics.

Since the KPDI Index was weighted on the basis of observed mortality in this dataset, we considered risk of hospitalisation as a separate outcome, censoring on death or end of follow-up, using nested Cox models. We found that the KPDI Index accounted for a proportion of the association between medication count and risk of hospitalisation.

Although polypharmacy and physiological frailty are associated with each other, our analyses suggest that confounding by indication does not explain very much of the association between medication count and risk of hospitalisation. VACS Index 2.0 is a widely validated, highly discriminating, physiological index that is predictive of hospitalisation and mortality among veterans with and without HIV.^12,21^ The updated version of the VACS Index we used in this analysis meets or exceeds the discrimination of previous indices validated among older individuals.^29^ Yet, full adjustment for the VACS Index, did not elucidate the association between polypharmacy and hospitalisation. After adjustments, polypharmacy remained independently associated with risk of hospitalisation in a dose–response manner.

After adjustment, the associations between medication count and risk of hospitalisation were strong for both people ageing with and without HIV, but was somewhat stronger for people ageing with HIV. This finding suggests that people ageing with HIV might have greater susceptibility to harm from polypharmacy due to higher order drug interactions, increased toxicity, or greater physiological frailty. Consistent with this finding is the observation that 5613 (61%) of 9186 people ageing with HIV in this analysis were on a boosted protease inhibitor regimen, which has been associated with increased rates of drug–drug interactions.^10,15^ Although the use of boosted regimens has decreased since 2009, a study in Madrid, Spain, that ran from Jan 31 to June 30, 2017, found that 23% of people ageing with HIV on antiretroviral medication were on a boosted regimen.^18^ Another possible reason for this increased susceptibility to harm due to polypharmacy is mitochondrial toxicity. All antiretrovirals included in ART have been shown to have some level of mitochondrial toxicity, as do many non-ART medications.^30–33^ Concern about mitochondrial toxicity is increasing in the ageing population generally, not just among HIV-positive populations.^30,34^ Finally, the average additional 10 years of polypharmacy for people ageing with HIV might cause low level, chronic toxic side-effects to become symptomatic.

Only a randomised trial can definitively resolve issues of confounding, but designing effective interventions without an understanding of underlying mechanisms is difficult. Our analyses offer stronger real-world evidence than previously reported that polypharmacy causes excess hospitalisation, partially through KPDIs. It also suggests that KPDIs are not the only reason for this excess risk.

Future work is needed to determine how best to proceed. Expert criteria such as START/STOPP^35^ or Beers^36^ were developed for individuals without HIV who are older than 65 years, and evidence for the effectiveness of these criteria in avoiding actual adverse health outcomes is contradictory and sparce. Although many studies in HIV-negative populations cite five medications as a critical threshold, no study has established a definitive threshold. Thresholds for injury probably depend on the specific regimen and associated drug interactions, the period of exposure, ongoing substance use, and the individual’s underlying physiological injury and genetic susceptibility.

ART regimens have evolved towards increased use of integrase strand transfer inhibitors and two-drug regimens, which will probably decrease the number of drug interactions and total medication count, so long as other non-ART medications are not added. People ageing with HIV on lifelong ART might continue to use alcohol and other substances that could interact with their medications and require special consideration. Sophisticated models will be needed, incorporating knowledge such as that contained in DrugBank, to account for what is known about drug–drug, drug–substance use, and pharmacogenetic interactions to aid the prescription of appropriate medications for these populations. Eventually, we will need to incorporate validated models into the clinical setting to help inform management of this complex problem.^37^

Our study has several strengths. The VACS has previously shown that the veterans without HIV in our sample are demographically, sociologically, and behaviourally similar to veterans living with HIV,^38^ thereby limiting the possibility that observed differences are due to these factors. We had direct access to pharmacy dispensing data in a national system with generous pharmaceutical coverage. Because patients might not fill all their prescriptions, data on medication orders (prescriptions) are less informative than dispensing data. We considered the association between polypharmacy and hospitalisation rather than potential drug interactions or other possible adverse effects. We compared health outcomes associated with polypharmacy among people ageing with HIV with demographically similar uninfected individuals. Our hospitalisation data are measured in a completely parallel manner among people ageing with and without HIV and include events occurring within the VA and care supported by other US Governmental sources (eg, Medicare and Medicaid).

Our study also has several limitations. The ART regimens that patients were undergoing reflect those in use in 2009. Use of this baseline was necessary for us to have adequate follow-up time for mortality endpoints that were used to weight the KPDI Index. Now that the methods and weighting have been developed, we plan to consider KPDIs among people ageing with and without HIV in more recent years. However, several studies from more recent periods suggest that people ageing with HIV continue to have more drug interactions than uninfected individuals.^18,27^ Our measures of medication count were conservative because they were based on medications dispensed within the VA and do not reflect outside prescriptions, over the counter, or complementary medications. Although people ageing with HIV in our sample were demographically similar to uninfected individuals, the strict 1:2 matching used in the VACS cohort was not preserved after the exclusions required for these analyses. The DrugBank database only reports KPDIs; it does not provide information concerning higher order (ie, greater than pairwise) interactions. We accounted for the severity of drug interaction by considering their average association with mortality and created a KPDI Index rather than using pre-existing weights included in DrugBank or other databases. In future, we hope to compare the explanatory power of our index against ones using pre-existing weights. Additionally, the nature of drug interactions is highly variable. We were not able to consider interactions with alcohol or other substances.^39^ We were not able to use any one of the many available approaches (including STOPP/START^35^ or Beers^36^) to determining potentially inappropriate medications. Furthermore, an in-depth consideration of each specific drug regimen was beyond the scope of this study. This is an observational analysis of real-world data and is subject to unadjusted confounding. Finally, our analysis should be replicated in health-care systems including a larger proportion of women to enable generation of more generalisable findings.

Accounting for demographics and physiological frailty, both people ageing with and without HIV have increasing risk of hospitalisation when exposed to increasing counts of non-ART medication. This risk is partially explained by KPDIs. The number of KPDIs increase dramatically with increasing medication counts, well beyond what would be expected by chance, especially for people ageing with HIV. Nonetheless, a substantial proportion of the risk associated with medication count is not explained by demographics, physiological frailty, or KPDI, especially for people ageing with HIV. Unknown drug interactions, including higher order interactions, substance–drug interactions, and increased susceptibility to cumulative toxicity might each have a role. Sophisticated informatics tools are needed to better guide drug selection and deprescribing to avoid excess hospitalisations and mortality due to polypharmacy.

## Supplementary Material

1

## Figures and Tables

**Table 1:** Baseline characteristics of sample overall and by HIV status

	Overall (n=47 116)	People ageing with HIV (n=9186)	People ageing without HIV (n=37 930)
**Age, years**			
<50	10 004 (21·2%)	2249 (24·5%)	7755 (20·5%)
50–64	30 413 (64·6%)	5683 (61·9%)	24 730 (65·2%)
≥65	6699 (14·2%)	1254 (13·7%)	5445 (14·4%)
**Sex**			
Female	1203 (2·6%)	196 (2·1%)	1007 (2·7%)
Male	45 913 (97·4%)	8990 (97·9%)	36 923 (97·4%)
**Race**			
White, non-Hispanic	19 309 (41·0%)	4012 (43·7%)	15 297 (40·3%)
Black, non-Hispanic	21 934 (46·6%)	4044 (44·0%)	17 890 (47·2%)
Hispanic	4167 (8·8%)	779 (8·5%)	3388 (8·9%)
Other or missing	1706 (3·6%)	351 (3·8%)	1355 (3·6%)
**Medical diagnoses**			
Hypertension, controlled, with treatment	20 567 (43·7%)	3496 (38·1%)	17 071 (45·0%)
Hypertension, uncontrolled	10 081 (21·4%)	1593 (17·3%)	8488 (22·4%)
Diabetes	15 304 (32·5%)	1996 (21·7%)	13 308 (35·1%)
Hyperlipidaemia	19 767 (42·0%)	3590 (39·1%)	16 177 (42·7%)
Coronary artery disease	4621 (9·8%)	669 (7·3%)	3952 (10·4%)
Chronic obstructive pulmonary disease	3233 (6·9%)	494 (5·4%)	2739 (7·2%)
Cirrhosis	551 (1·2%)	163 (1·8%)	388 (1·0%)
Chronic pain	22 826 (48·5%)	3332 (36·3%)	19 494 (51·4%)
Gastro-oesophageal reflux disease	5126 (10·9%)	631 (6·9%)	4495 (11·9%)
Obesity (BMI ≥35 kg/m^2^)	7850 (16·7%)	444 (4·8%)	7406 (19·5%)
**Psychiatric diagnoses**			
Major depression	3592 (7·6%)	771 (8·4%)	2821 (7·4%)
Bipolar disorder	2824 (6·0%)	483 (5·3%)	2341 (6·2%)
Post-traumatic stress disorder	6529 (13·9%)	735 (8·0%)	5794 (15·3%)
Schizophrenia	2640 (5·6%)	211 (2·3%)	2429 (6·4%)
Any psychiatric disorder	13 004 (27·6%)	1858 (20·2%)	11 146 (29·4%)
**Substance use**			
Alcohol-related diagnosis	5048 (10·7%)	793 (8·6%)	4255 (11·2%)
Drug abuse and dependence	4692 (10·0%)	1072 (11·7%)	3620 (9·5%)
Alcohol or drug diagnoses	7105 (15·1%)	1360 (14·8%)	5745 (15·2%)
Current smoker	23 527 (49·9%)	4827 (52·6%)	18 700 (49·3%)
Former smoker	8622 (18·3%)	1519 (16·5%)	7103 (18·7%)
**VACS Index score and components**			
Total score	34 (27–43)	48 (39–58)	32 (26–39)
Age, years	56 (51–62)	56 (50–61)	56 (51–62)
HIV-1 RNA, copies per mL	NA	<400	NA
CD4 count, cells per mm3	NA	511 (346–720)	NA
Haemoglobin, dL	14 (13–15)	14 (13–15)	14 (14–15)
Fibrosis-4 score	1·19 (0·89–1·66)	1·38 (1·00–1·97)	1·15 (0·87–1·59)
Estimated glomerular filtration rate, mL/min per 1·73 m^2^	88 (73–102)	88 (73–104)	88 (73–102)
White blood cell count, ×10^9^ per L	7 (5–8)	6 (5–7)	7 (6–8)
Albumin, g/dL	4·1 (3·9–4·4)	4·1 (3·8–4·5)	4·1 (3·9–4·4)
BMI, kg/m^2^	29 (25–33)	26 (23–29)	30 (26–34)
Hepatitis C infection	9085 (19·3%)	3163 (34·4%)	5922 (15·6%)
**Medication classes**			
ACE inhibitors	15 625 (33·2%)	2389 (26·0%)	13 236 (34·9%)
β blockers	13 301 (28·2%)	1989 (21·7%)	11 312 (29·8%)
Diuretics	9718 (20·6%)	1388 (15·1%)	8330 (22·0%)
Calcium-channel blockers	10 990 (23·3%)	1297 (14·1%)	9693 (25·6%)
Lipid-lowering agents	23 861 (50·6%)	3855 (42·0%)	20 006 (52·7%)
Oral glucose-lowering agents	9326 (19·8%)	980 (10·7%)	8346 (22·0%)
Gastric medications	14 184 (30·1%)	1766 (19·2%)	12 418 (32·7%)
Antidepressants	14 664 (31·1%)	2937 (32·0%)	11 727 (30·9%)
Non-opioid analgesics	12 222 (25·9%)	1722 (18·8%)	10 500 (27·7%)
Genitourinary agents	9435 (20·0%)	1797 (19·6%)	7638 (20·1%)
**Outcomes**			
Hospitalisaton	25 846 (54·9%)	5609 (61·1%)	20 237 (53·4%)
All-cause death	10 881 (23·1%)	2329 (25·4%)	8552 (22·6%)

Data are n (%) or median (IQR). ACE=angiotensin-converting enzyme. BMI=body-mass index. NA=not applicable. VACS=Veterans Aging Cohort Study.

**Table 2:** Association between KPDI Index score, non-ART medication count, VACS Index 2.0 score, and all-cause mortality rates in people ageing with and without HIV

	n	KPDI index score	Non-ART medication count	VACS Index 2.0 score	All-cause deaths per 1000 person-years (95% CI)
**People ageing with HIV**					
No KPDIs	473	0 (0)	1·09 (0·82)	49 (15)	27 (22 to 32)
KPDI Index quintile 1	1161	–0·43 (0·43)	4·57 (2·38)	48 (14)	22 (20 to 26)
KPDI Index quintile 2	3688	0·00 (0·03)	2·15 (1·58)	47 (14)	21 (20 to 23)
KPDI Index quintile 3	2010	0·34 (0·19)	4·34 (2·22)	52 (15)	37 (34 to 40)
KPDI Index quintile 4	1263	1·45 (0·54)	6·56 (2·55)	54 (15)	47 (43 to 51)
KPDI Index quintile 5	591	5·02 (2·34)	9·01 (2·87)	57 (16)	63 (56 to 71)
**People ageing without HIV**					
No KPDI	7793	0 (0)	1·20 (0·90)	31 (11)	17 (16 to 18)
KPDI Index quintile 1	5832	–0·44 (0·45)	4·54 (2·40)	32 (10)	15 (14 to 16)
KPDI Index quintile 2	6816	0·01 (0·05)	2·91 (1·83)	32 (10)	19 (18 to 20)
KPDI Index quintile 3	8503	0·35 (0·19)	4·45 (2·20)	34 (10)	27 (26 to 28)
KPDI Index quintile 4	5816	1·47 (0·54)	6·69 (2·50)	36 (11)	41 (39 to 43)
KPDI Index quintile 5	3170	5·46 (3·18)	9·36 (2·62)	39 (12)	76 (72 to 79)

Data are n, mean (SD), or mean (95% CI). ART=antiretroviral therapy. KPDI=known pairwise drug interaction. VACS= Veterans Aging Cohort Study.

**Table 3:** Nested survival models of medication count and risk of hospitalisation

	Unadjusted	Progressively adjusted for additional factors		
		
		Adjusted for demographics	Adjusted for demographics and severity of illness	Adjusted for demographics, severity of illness, and KPDI Index
**People ageing with HIV (n=9186)**				
Non-ART medication count	1·08 (1·07–1·09)	1·08 (1·07–1·09)	1·07 (1·06–1·08)	1·06 (1·05–1·07)
Age per 10 years	··	1·13 (1·10–1·16)	0·96 (0·93–0·99)	0·96 (0·93–0·99)
Sex				
Female	··	1·13 (0·94–1·34)	1·07 (0·89–1·27)	1·07 (0·90–1·28)
Male	1 (ref)	1 (ref)	1 (ref)	1 (ref)
Race				
White, non-Hispanic	1 (ref)	1 (ref)	1 (ref)	1 (ref)
Black, non-Hispanic	··	1·27 (1·20–1·34)	1·16 (1·10–1·23)	1·17 (1·10–1·24)
Hispanic	··	1·16 (1·05–1·28)	1·11 (1·01–1·22)	1·11 (1·01–1·23)
Other	··	0·72 (0·61–0·85)	0·73 (0·62–0·86)	0·74 (0·63–0·87)
VACS Index 2.0 per 5 points	··	··	1·11 (1·10–1·12)	1·11 (1·10–1·12)
KPDI Index score				
No KPDI	··	··	··	1·10 (0·97–1·25)
KPDI Index quintile 1	··	··	··	1·03 (0·94–1·13)
KPDI Index quintile 2	··	··	··	1 (ref)
KPDI Index quintile 3	··	··	··	1·12 (1·04–1·21)
KPDI Index quintile 4	··	··	··	1·14 (1·04–1·26)
KPDI Index quintile 5	··	··	··	1·12 (0·98–1·28)
**People ageing without HIV (n=37930)**				
Non-ART medication count	1·08 (1·07–1·08)	1·07 (1·07–1·08)	1·07 (1·06–1·07)	1·04 (1·03–1·05)
Age per 10 years	··	1·00 (0·99–1·02)	0·83 (0·81–0·84)	0·82 (0·81–0·84)
Sex				
Female	··	0·91 (0·83–0·99)	0·83 (0·76–0·91)	0·85 (0·77–0·92)
Male	1 (ref)	1 (ref)	1 (ref)	1 (ref)
Race				
White, non-Hispanic	1 (ref)	1 (ref)	1 (ref)	1 (ref)
Black, non-Hispanic	··	1·29 (1·25–1·33)	1·24 (1·20–1·28)	1·26 (1·22–1·29)
Hispanic	··	1·07 (1·02–1·13)	1·11 (1·05–1·16)	1·12 (1·06–1·17)
Other	··	0·57 (0·52–0·63)	0·59 (0·54–0·65)	0·60 (0·54–0·66)
VACS Index 2.0 per 5 points	··	··	1·15 (1·15–1·16)	1·15 (1·14–1·16)
KPDI Index score				
No KPDI	··	··	··	0·88 (0·84–0·93)
KPDI Index quintile 1	··	··	··	0·96 (0·91–1·01)
KPDI Index quintile 2	··	··	··	1 (ref)
KPDI Index quintile 3	··	··	··	1·10 (1·05–1·15)
KPDI Index quintile 4	··	··	··	1·22 (1·15–1·28)
KPDI Index quintile 5	··	··	··	1·34 (1·25–1·43)

Data are hazard ratios with 95% CIs in parentheses. ART=antiretroviral therapy. KPDI=known pairwise drug interaction. VACS= Veterans Aging Cohort Study.

## Research in context

### Evidence before this study

We did a related article search in PubMed on July 23, 2021, using a review of polypharmacy and HIV (PMID 31833963). This search yielded 98 articles published in the period 2004–21, a pool that was then restricted to just articles on HIV or with HIV in the title or abstract, which left 34 articles. We reviewed the abstracts of these articles and found that 20 addressed polypharmacy among individuals with HIV. These articles reported data from North America, Europe, and Australia. Six articles considered the association of medication count with a clinical outcome (falls or fractures, discontinuation or non-adherence to antiretroviral treatment, and non-fatal opioid overdose). Only our previous publication considered associations with admission to hospital (ie, hospitalisation) and mortality. Most studies found a substantial prevalence of polypharmacy, which increased with increased age. Several studies showed increasing prevalence of polypharmacy among people ageing with HIV over calendar time. Ten articles considered the association of medication count with potentially inappropriate medications. These studies showed that potentially inappropriate medications and antiretroviral therapy (ART)-non-ART medication interactions increase with increasing medication count. Five previous studies compared polypharmacy frequency by HIV status, which found that, controlling for age, polypharmacy was more common among people with HIV infection.

### Added value of this study

We used large scale, real-world data to consider how much of the association between polypharmacy and hospitalisation might be explained by known pairwise drug interactions not restricted to ART-non-ART interactions. We used an updated and validated measure of physiological frailty to account for confounding by indication. No previous study has considered the association between drug-drug interactions among people ageing with HIV and hospitalisation, compared this association with that observed for uninfected individuals, or considered how much of the association between polypharmacy and hospitalisation is explained by established drug-drug interactions.

### Implications of all the available evidence

Polypharmacy is an increasing problem worldwide, with particularly important implications for those ageing with HIV. In future, we hope to apply our index of known pairwise drug interactions to more recent data (ie, from the past 5 years) to determine whether the differences between people ageing with and without HIV remain. Regardless, providers should obtain a complete list of medications, consider non-pharmacological treatments where feasible, and select non-ART medications to minimise polypharmacy and associated drug-drug interactions. Where possible, electronic decision support might greatly facilitate these efforts.
