# Supplementary material for: Polypharmacy-associated risk of hospitalisation among people ageing with and without HIV: an observational study
Source: Lancet Healthy Longev. Author manuscript; Available in PMC 2021 Dec 2. (PMC8639138; doi:10.1016/S2666-7568(21)00206-3)
Supplement: 1 [file NIHMS1744542-supplement-1.pdf]

THE LANCET
Healthy Longevity

Supplementary appendix

This appendix formed part of the original submission and has been peer reviewed. We post it as supplied by the authors.

Supplement to: Justice AC, Gordon KS, Romero J, et al. Polypharmacy-associated risk of hospitalisation among people ageing with and without HIV: an observational study. *Lancet Healthy Longev* 2021; **2:** e639–50.

Appendix

Study Overview

We conducted a prospective analysis of the Veterans Aging Cohort Study (VACS). Briefly, VACS is a cohort of PAH and uninfected individuals matched 1:2 on age, sex, race/ethnicity and site of care identified from the United States Veterans Health Administration (VA) administrative data. Baseline was established in fiscal year (FY) 2009 (October 1st 2008 to September 30th 2009). Data were obtained from the VA corporate data warehouse and included demographic characteristics, hospital and outpatient diagnoses (recorded using International Classification of Diseases, Ninth Revision [ICD-9] codes), laboratory results, and dispensed medications from the Pharmacy Benefits Management (PBM) Program. Center for Medicare and Medicaid Services (CMS) claims data were available for Veterans also enrolled in this program and were merged with VACS data. Patients were followed five years through September 30, 2015 in our original paper. In this current work, patients were followed to March 31, 2019.

Study Population

We included PAH receiving ART and uninfected comparators who were receiving at least one prescription medication from the Veterans Affairs Healthcare System (VA) in fiscal year 2009. This year was selected for the original study to allow five years of follow-up after establishing the exposure to polypharmacy. PAH were considered on ART if they were prescribed three or more antiretroviral agents, excluding low dose ritonavir. We required uninfected individuals to be on at least one VA dispensed medication to ensure that they were obtaining medications from the VA. Among patients

alive at the end of FY2009, we excluded patients who had: 1) any cancer diagnosis except non-epithelial skin cancer (n=1,874) as their severity of illness may not be adequately reflected in the VACS Index; 2) ambiguous HIV status (n=99) to make a clear comparison between PAH and uninfected individuals; 3) PAH who were not virally suppressed (>400 HIV-1 RNA copies/mL) in the last 6 months of baseline FY2009 (n=9,018) as we sought to study polypharmacy among those on successful ART; 4) insufficient data to calculate VACS Index (n=4085); and 5) on more than 15 medications to eliminate highly leveraged observations (n=534, 1% of the analytic sample).

Medication Exposure

We determined receipt of all outpatient preparations (i.e. oral, inhaled, topical, or injectable) of medications dispensed through the VA using prescription pharmacy fill/refill data. We excluded prescriptions classified as diagnostic supplies (e.g. glucose test strips); emollients; eye washes and lubricants; soaps, shampoos and soap-free cleaners; mouthwashes; sun protectants and screens; irrigation solutions; ceruminolytics; deodorants, and antiperspirants; and contact lens solutions from analyses. Our analysis considered medications prescribed and refilled in 2009 allowing for a 30 day refill window, consistent with previous definitions.(1)

Days of medication receipt were calculated based on prescription information, assuming the prescription was taken as directed. Medications were categorized according to VA class. We calculated the mean number of unique non-ARV chronic medications received by each patient during FY2009 by summing the number of days supplied for each medication and dividing the total by 365 days. Each component of co-formulated medications was counted separately.

Health Outcomes

Hospitalization was identified from the VA (including fee based) and Center for Medicare and Medicaid Services (CMS). All-cause mortality was obtained from the VA Vital Status File, which includes data from inpatient records, the VA Beneficiary Identification Records Locator Subsystem (BIRLS), Social Security Administration, and CMS. Excellent reliability and validity of the Vital Status File has been established by comparison with the National Death Registry. (2) We conducted surveillance for hospitalization and mortality; time to event was calculated from October 1, 2010 to the date of first event or censored on March 31, 2019.

Covariates

Demographic variables included age, race/ethnicity and gender. Hepatitis C virus (HCV) status was considered positive if a patient had a positive HCV antibody test or HCV RNA or ICD-9 codes for HCV infection.

REFERENCES

1. Edelman EJ, Gordon K, Becker WC, Goulet JL, Skanderson M, Gaither JR, et al. Receipt of Opioid Analgesics by HIV-Infected and Uninfected Patients. J Gen Intern Med. 2012.

2. Cowper DC, Kubal JD, Maynard C, Hynes DM. A primer and comparative review of major US mortality databases. Ann Epidemiol. 2002;12(7):462-8.
